# First person – Elysabeth Otte

**DOI:** 10.1242/dmm.053231

**Published:** 2026-10-28

## Abstract

First Person is a series of interviews with the first authors of a selection of papers published in Disease Models & Mechanisms, helping researchers promote themselves alongside their papers. Elysabeth Otte is first author on ‘ Cognitive flexibility deficits differ between the sexes in a transchromosomic model of Down syndrome’, published in DMM. Elysabeth is a PhD candidate in the lab of Randall J. Roper, PhD at Indiana University Indianapolis, Indianapolis, IN, USA, investigating the mechanisms underlying neural development and behaviour, and how studying behaviour can provide insights into human health and disease.

**Figure:**
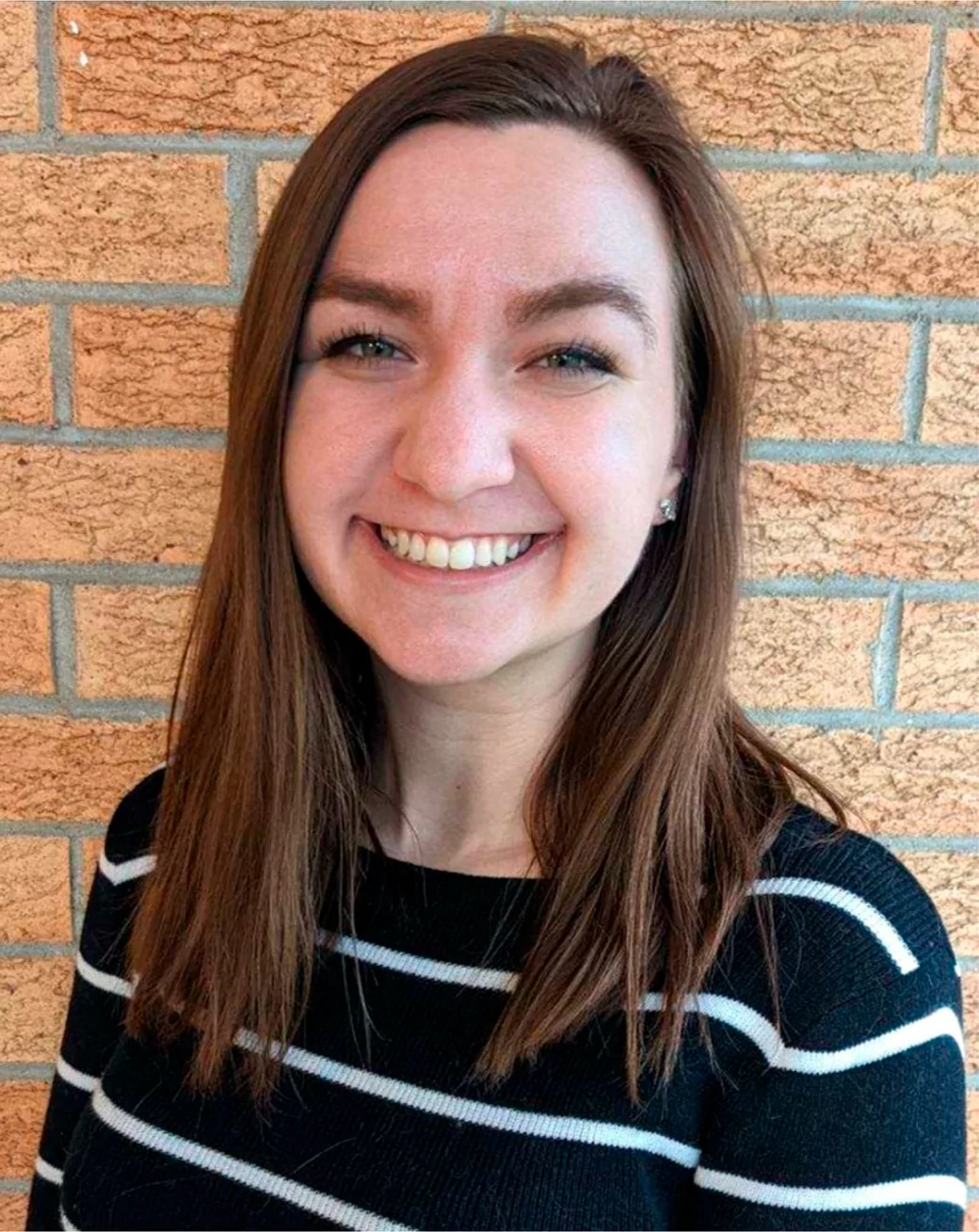
Elysabeth Otte

**Who or what inspired you to become a scientist?**

I have wanted to be a scientist for as long as I can remember. As a child, I was curious about the world and wanted to understand how things worked. I remember using a little microscope to look at water samples from a creek by my house and comparing them to samples from my own fish tank, and I remember following my pets around our house and documenting their behaviours – things like ‘ate a snack at 11:10’ and ‘went into kitchen at 14:04’. I don't think an individual event or person led me to science – just a natural curiosity and observational mindset that made me want to spend my life formulating and answering questions.

**What is the main question or challenge in disease biology you are addressing in this paper? How did you go about investigating your question or challenge?**

The main challenge we are addressing in our paper is understanding how sex influences the cognitive and behavioural phenotypes associated with Down syndrome, while also characterizing a new model that incorporates triplicated human, rather than mouse, genes. We investigated this by comparing male and female transchromosomic mice with euploid littermates across a range of behavioural assays designed to assess learning, memory and cognitive flexibility.

Our study found that mice with extra copies of human genes found on human chromosome 21 show differences between males and females in their ability to learn, remember information and adapt to changing conditions

**How would you explain the main findings of your paper to non-scientific family and friends?**

Our study found that mice with extra copies of human genes found on human chromosome 21 show differences between males and females in their ability to learn, remember information and adapt to changing conditions. These findings could help us better understand why people with Down syndrome can have different learning and behavioural experiences.

**What are the potential implications of these results for disease biology and the possible impact on patients?**

These results could contribute to a better understanding of how sex may influence the cognitive and behavioural characteristics of Down syndrome, and may ultimately help inform research into the differing needs of individuals with Down syndrome. They also emphasize the importance of including both sexes in studies of Down syndrome so that potential differences are not overlooked.

**Figure:**
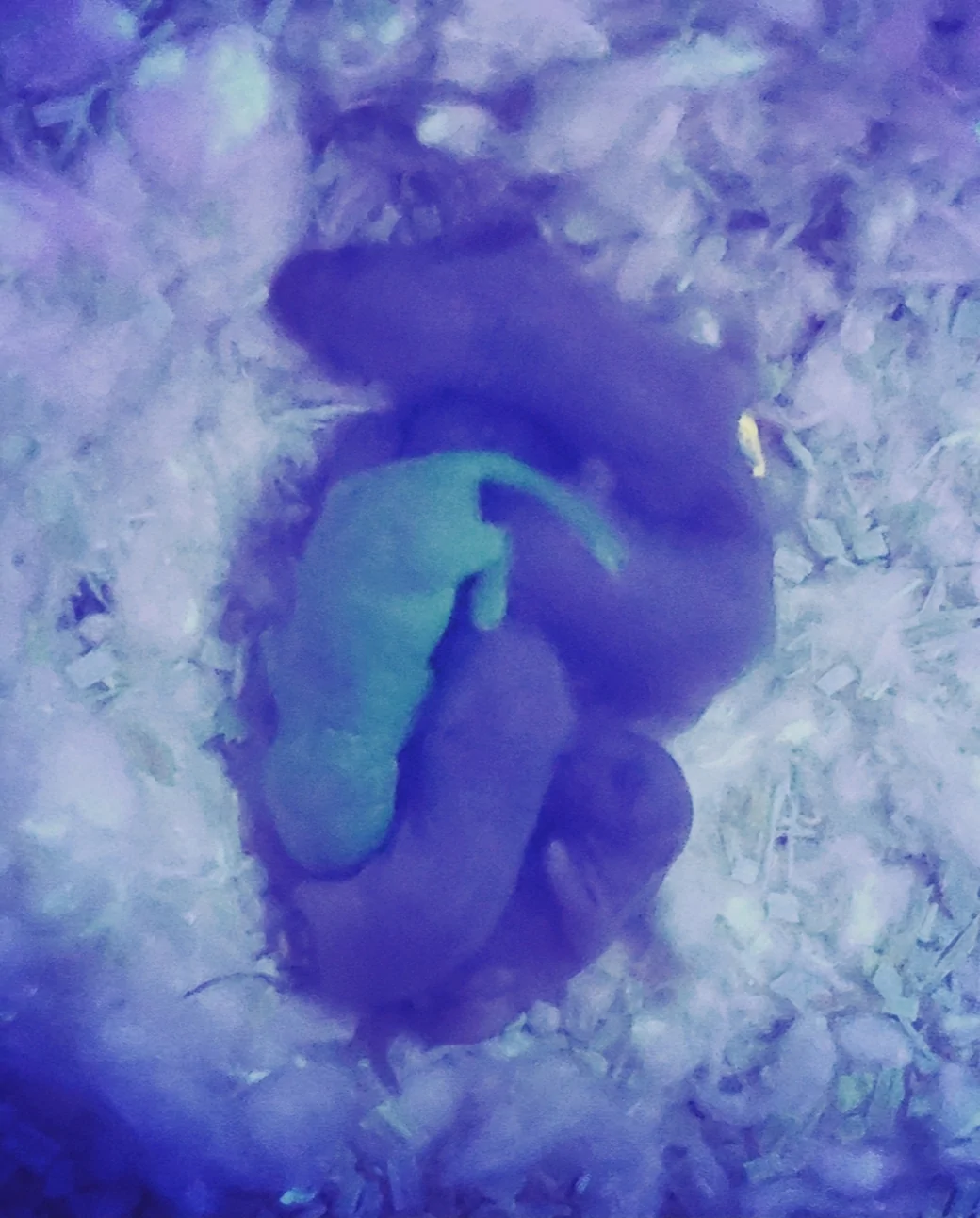
Our first successfully bred litter of TcMAC21 mice, including a GFP-expressing trisomic pup.

**Why did you choose DMM for your paper?**

We chose DMM for this paper because of its reputation for connecting research with clinical applications, and because of its publication history of highlighting research that provides novel insights into human disease.

If you're the smartest, most qualified person in the room, then you're in the wrong room

**Given your current role, what challenges do you face and what changes could improve the professional lives of other scientists in this role?**

As a PhD candidate, I face challenges associated with being a trainee and with always being one of the less-experienced people in the room. This can be difficult when it comes to applying for positions and trying to make connections with well-established researchers, but I also like to see it as an opportunity. If you're the smartest, most qualified person in the room, then you're in the wrong room.

**What's next for you?**

I'm hoping to finish some additional studies in the near future before moving on to a postdoctoral position. I'm excited to continue developing my research interests while gaining experience in a new research environment.

**Tell us something interesting about yourself that wouldn't be on your CV**

Something that wouldn't be on my CV is my soft spot for animals. Over the years, I've cared for and rescued many wild animals, including a pet opossum named Ickity and a pigeon named Hercules. I regularly take in foster and hospice animals that need a place to stay, and I've always had a hard time saying no to animals in need.
